# Instant prediction of scientific paper cited potential based on semantic and metadata features: Taking artificial intelligence field as an example

**DOI:** 10.1371/journal.pone.0312945

**Published:** 2024-12-02

**Authors:** Hou Zhu, Li Shuhuai

**Affiliations:** 1 School of Information Management, Sun Yat-sen University, Guangzhou, China; 2 Australian Artificial Intelligence Institute, University of Technology Sydney, Sydney, NSW, Australia; 3 Credit Card Center of China Guangfa Bank Co., Ltd, Guangzhou, China; Vellore Institute of Technology, INDIA

## Abstract

With the continuous increase in the number of academic researchers, the volume of scientific papers is also increasing rapidly. The challenge of identifying papers with greater potential academic impact from this large pool has received increasing attention. The citation frequency of a paper is often used as an objective indicator to gauge the academic influence of the paper. The task of citation frequency prediction based on historical citation data in previous studies can achieve high accuracy. However, it can only be executed after the paper has been published for a period. The delay is not conducive to timely discovery of papers with high citation frequency. In this paper, we propose a novel method for predicting cited potential of a paper based on the metadata and semantic information, which can predict the cited potential of academic paper instantly once it has been published. Specifically, the semantic information, such as abstract, semantic span and semantic inflection, is extracted to enhance the ability of the prediction model based on machine learning. To prove the effectiveness and rationality of cited potential prediction model, we conduct two experiments to validate the model and find the most effective combination of input information. The empirical experiments show that the prediction accuracy of our proposed model can reach 88% for the instant prediction of citation.

## 1. Introduction

With the development of science, the continuous increase in the number of scholars has led to a sharp rise of academic papers. Finding the most valuable papers from a large number of academic papers has become an inevitable challenge for scientific researchers, especially when it comes to identifying those papers that may have a higher impact in the future, despite their short publication time and low citation frequency. Therefore, the evaluation of the scientific papers’ impact has become an important research focus.

At present, there are many indicators for evaluating the influence of scientific papers. Among these indicators, the most widely used indicator is the citation frequency. Generally speaking, the paper with higher citation frequency is considered to be more influential. Researchers have also proposed many classic indicators based on the number of citations to evaluate the influence of scholars, journals and academic papers [1–4], such as h-index [5], impact factor [6]. In addition, in order to obtain more citations in future research work and improve influence, researchers tend to focus on how many citations their research papers will get in the future and the factors that affect the citations of their research papers. The existing paper citation prediction methods are usually based on the accumulated citations of the paper, such as using the citations accumulated within the first five years to predict its future citations [7]. Although these methods’ prediction accuracy rate is high, they are limited to predicting citations of already-published papers and have a lag in the prediction time, which means that these models can’t predict the citations instantaneously. The previous models used the existing citations number of a paper as one of the inputs to predict the future citations of the paper. Obviously, these models can’t predict the number of citations for a newly published paper because there is no existing citations number yet.

If the citations of the paper can be predicted in advance to assess the possible influence of the paper, researchers can quickly discover high-impact papers. Simultaneously, this approach can relieve the pressure of scholars to retrieve and process paper data, allowing them to put the saved time and energy into other scientific research activities [8, 9]. In addition, the researchers can also identify in advance which research will attract more attention from the society in the future, enabling them to better plan their research direction. Therefore, the prediction of the citations of papers can not only help researchers to discover potential high-impact papers, but also help authors to allocate resources, which is a task with important application value.

In this paper, we propose a model for predicting the cited potential of a scientific paper based on its metadata and semantic information. In other words, the proposed model takes the metadata and semantic information of a paper, and then predicts whether its citation frequency can become high-level or not.

In the terminology of deep learning methods, we considered the citation prediction as a classification learning problem, and then we utilized neural networks as a powerful model for learning the prediction task. We designed a customized model which is appropriate for learning the metadata and semantic information. We also conducted comprehensive experiments to show the reasonableness and effectiveness of the proposed model.

The rest of this paper is organized as follows: In Section 2 we review the related work. In Section 3 we formulate the problem and present the proposed model. Section 4 describes the prepared dataset which is utilized in our experiments. In Section 5 we evaluate the proposed method. Finally, we conclude the paper in Section 6 and describe the future works.

## 2. Related work

Academic papers are the most important output of scientific research. Accurate evaluation of academic papers has always been one of the core issues of Scientometrics. The influence of academic papers refers to the changes in readers’ knowledge and cognition caused by the information carried by academic papers in a certain way [10]. For a long time, the methods of measuring the impact of academic papers are qualitative evaluation based on peer review and quantitative evaluation based on bibliometric indicators. Among them, qualitative evaluation based on peer review is greatly affected by the subjective judgment of the evaluation subject. Different evaluation subjects may affect the objectivity and fairness of the final evaluation results due to factors such as their own research direction, knowledge system [11]. At the meanwhile, peer review requires more expert participation and takes more time. So the cost of peer review is often higher than the evaluation method using bibliometric indicators. As a result, the evaluation based on bibliometric index is widely used because of its efficient and objectivity in evaluating the academic influence of papers.

Bibliometric indicators mainly include citation frequency, impact factor, characteristic factor and paper impact score [12]. Among these indicators, the most simple, effective indicator is the citation frequency of scientific papers. The paper’s citation frequency has been widely used in the evaluation of the paper’s impact. Bai et al. predict citation frequency of a paper based on the number of citations accumulated after the paper was published and the academic influence of scholars who cited the paper [13]. Zhao et al. construct a comprehensive evaluation model for the influence of academic papers based on alternative metrology indicators [14]. Therefore, the use of paper citations as a reflection of the paper’s impact has been widely recognized in academia. So in our paper, the citations are also used as a objective reflection of the paper’s influence. However, once a paper is published, factors such as the research field, authors, and even the publication in which it is published will have an impact on the number of citations of the paper [15]. Hence, the research on predicting the number of citations of papers needs to comprehensively consider various influencing factors.

At present, the information used to predict the citations can be divided into two parts, the first is based on traditional bibliometric indicators and alternative quantitative indicators [16], such as the length of the papers [17], the number of authors [18, 19], the cooperation network [20], the accumulated citations [8, 21] and so on. The second is to predict the citations of the paper from the perspective of the semantic information of the paper [22, 23].

Researchers usually pay more attention to scientific papers published in the last 2 years and the more references a paper has, the higher its citation frequency will be [24]. Chakraborty et al. point out that the citation frequency of papers have a Matthew effect within a short time after its publication, that is, papers with more citations accumulated in short time will gain more citations in the future [25]. Abrishami et al. propose a model using deep neutral network to predict the citation frequency based on n its citations during the early years of publication [9]. Xiong et al. find a significant correlation between the number of downloads in the early stage of publication and the number of citations in the later stage of the paper, which can be used as one of the basis for predicting the citations in the later stage of the paper [26].

Among those indicators, bibliometric indicators can be divided into three categories: paper-related, author-related and journal-related. In terms of paper-related indicators, there is a significant correlation between the paper type and the citations. The citation frequency of review papers are often higher than research papers [27, 28]. In regard of the publication time, papers with faster citation accumulation rate will have higher citations in the future [29]. There are also significant differences of citations according to different disciplines. For example, the h-index of scholars is significantly correlated with the disciplines, and the h-index of scholars in the field of life sciences is significantly higher than that of scholars in the field of physics [5]. Roth et al. evaluate the impact of papers based on the dynamic citation network, and find that the newer the references cited in the paper, the higher the probability of the paper being cited in the future [30]. The title is an important part of an academic paper, and a suitable title can attract the attention of subsequent researchers by accurately reflecting the research topic of the paper. Habibzadeh et al. analyze the correlation between the length of the title and the number of citations based on the method of linear regression [31]. In their dataset, longer titles have higher citations, especially in journals with high Journal Impact Factor (JIF). Jamali et al. firstly explore the relationship between title types and the citation of papers [32]. They study three main title types of PLoS journal papers—descriptive titles, conclusive titles, and question titles. The results show that the question title is more likely to attract the initial attention of researchers, and its papers are more downloaded but relatively less frequently cited, while papers with descriptive or declarative titles are more likely to be cited. Sohrabi et al. explore the frequency of the appearance of keywords in the abstract and the journal and the citation frequency of the paper [33]. The results show that the frequency of keywords in the abstract is positively correlated with the citation frequency of the paper.

In terms of author-related metadata, the relationship between the size of the author team and its influence has been widely concerned by researchers. Most of them believe that the two are positively correlated, that is, the size of the author team is significantly correlated with citations. The papers with larger size of the author team will have higher citation frequency [19]. Different authors have different influences on the citation frequency of their papers because of their academic influence. High-level authors often have higher academic output and academic influence, and play a very important role in improving the academic level and academic influence of papers [34]. In the citation prediction system established by Yan et al., h-index of the author is positively correlation with the citation frequency [35]. Tahamtan et al. emphasize that the authors with the higher citation frequency accumulated in history are more likely to be cited in the future [36]. Chakraborty et al. find that authors who published more articles in the past will gain more citations in the future, and so as the diversity of the author’s publishing field [25]. In addition, the author’s institution, ethnic diversity will also have a significant positive impact on the citation frequency of papers. Uzzi et al. point out that international collaborative papers are easy to obtain higher citation frequency, and interdisciplinary cooperation will promote the increase of citations of papers [37]. Author diversity such as discipline, gender, ethnicity, institution and academic age also has a positive impact on paper’s citation [38].

In terms of journal-related metadata, there is a positive interaction between journal influence and the citation of papers. Journals which publish a large number of highly cited paper will gain greater influence in the future [39]. Furthermore, papers published in core journals are more likely to be cited for the first time within one to two years of publication than papers published in non-core journals [40].

Semantics is an important factor in determining the citation of a paper. The semantics of scientific papers provide valuable information for the papers’ citation [22]. With the development of big data analysis technology, researchers have begun to gradually incorporate the semantic information of scientific papers into the citation prediction model. The previous research on the citation frequency prediction of scientific papers based on the semantics is mainly carried out from two perspectives: the semantics of the paper itself and the semantics of peer-reviewed texts. A scientific paper usually contains research questions, research methods and research results, and only through this information can subsequent researchers be able to determine the research value of the academic paper. The semantics of the paper itself contains all the content of the paper research, and the title and abstract are also important parts to obtain valuable information of a paper [22], which play an important role in attracting researchers to read the paper, thereby affecting the number of citations [22, 31, 41]. Letchford et al. point out that the frequency of common words in the abstract is inversely correlated with the citation of the paper [41]. Toubia et al. explore the influencing factors of the citation of papers from the perspective of paper writing style, and propose that three factors, the semantic span between sentences in scientific papers, the average amount of information covered by sentences, and the degree of semantic transition between sentences, affect the citation frequency of papers [42]. Ma et al. use Recurrent Neural Network (RNN) to extract features from the semantic information contained in abstracts, and combine with the citations during the early years of publication to effectively predict the future citation frequency of the paper [7]. With the opening of the peer review process, textual information such as peer-reviewed texts also has been used in research to improve the effectiveness of citation prediction models. Wang et al. use peer-reviewed text information to predict whether a paper could be published [43]. Li et al. conduct semantic model of peer-reviewed texts and integrate factors such as topic distribution and author influence to construct and expand the independent variables of the model [23]. The results show that peer-reviewed texts can effectively predict the citation frequency of papers.

It is worth noting that there has been a lot of research to predict the citation of scientific paper, but these researches can’t realize instant prediction. In this paper, we use papers published from 2011 to 2013 to predict the citation of papers published in 2015. This is the biggest difference of input information between our research and recent other studies since it does not utilize short-term-citation frequency to predict the citations of scientific papers, and therefore the results are applicable and instant when the input information is limited.

## 3. Methodology

In this section, we formulate the problem and describe our proposed method along with the details of the employed techniques.

### 3.1 Problem statement

According to previous research, there is a significant correlation between the specific metadata of the paper, such as the number of authors, h-index, etc., and the number of citations of the paper [19]. In addition, the semantic information [25] involved in the paper is also related to the citations of the paper. In addition to the semantic information contained in the content of the paper, Toubia et al. propose the semantic span and semantic inflection index to quantify the writing style of the paper, which provide a supplement to the semantic information of the paper content [42]. Therefore, this paper will propose an instant prediction model which aims to identify whether a paper’s citation can become high-level or not once it is accepted by a journal. This study will integrate the semantic information which include title, abstract and writing style and metadata of papers to predict whether a paper’s citation will become high-level.

### 3.2 Implementation progress

This study is expected to conduct two experiments. Experiment 1 is a comparison experiment to verify whether the metadata, semantics are able to predict citations respectively or not and identify the algorithm with better text classification effect. The dataset used in Experiment 1 is composed of the journal papers published from 2011 to 2015. The dataset is divided into high-level cited papers and non-high-level cited papers and splits into training set and test randomly. And this study designs three different inputs of metadata, paper semantics, metadata and paper semantics to respectively predict whether the papers’ citation will become high-level or not within five years after published, the comparison experiments of different combinations of models are shown in Table 1.

**Table 1:** The parameters of comparison experiment and instant experiment in this research.

	Input	Purpose	
Comparison Experiment	Title, Abstract	Can semantic information predict high-level cited paper?	Verify the validity of the model
	Title, Abstract, Writing Style		
	Metadata	Can metadata predict high-level cited paper?	
	Title, Abstract, Writing Style, Metadata	Can semantic information and metadata predict high-level cited paper?	
Instant Experiment 2011,2012,2013 →2015	Title, Abstract, Writing Style, Metadata	Verify the effectiveness of the model	

In the practical application of instant prediction of high-level cited papers, the object of prediction, that is, the test set, should be the papers to be published in the latest year, and the training set should only contain the citation frequency up to the latest year. However, considering that the division of high-level and non-high-level cited papers in Experiment 1 requires a prior assumption to know the five-year citations of the paper both in training set and test set, and the division criteria of the training set and the test set of Experiment 1 does not take into account of the publication year, that is there is a phenomenon of predicting citations of papers published in 2011 by papers published in 2015. Therefore, the results of Experiment 1 have great limitations in practical application. In order to improve the effectiveness of the model in practical applications, this study considers using the model to conduct instant prediction experiments on the paper dataset, that is, Experiment 2, as shown in Table 1. Experiment 2 selects the model with better text classification effect in Experiment 1, and predicts the citation of papers published in 2015 through the papers published from 2011 to 2013. The citation database will provide papers’ citation frequency every per year after it is published, which will include the citations of the paper in the year it was published. However, the citations of papers published in the same year will also be greatly affected by the publication time, so the training set of Experiment 2 is consisted of the papers that have been published at least two years before 2015.

According to the difference between the two experiments, the classification standard of high-level cited paper of the two experiments are as follows.

Firstly, for Experiment 1, the citations of the papers within five years are obtained and sorted in descending order, and the citations within five years in the top third is considered as high-level, and the remaining two-thirds are considered to be non-high-level. The combination of the two parts constitutes the dataset of Experiment 1.

Secondly, the definition of high-level based on five-year citations in Experiment 1 is difficult to achieve in the practical application of the instant prediction task of high-level cited papers. In order to verify the effectiveness of our model in practical applications, in Experiment 2 the high-level cited papers in the training set are defined as the papers published in the same year, whose citation frequency accumulated from the year of publication to the year of 2015 rank in the top third of the dataset are high-level. The remaining two-thirds is non-high-level. For example, the citations of paper A are high-level just because in all papers published in 2011, A’s cumulative citation frequency for four years from 2011 to 2014 ranks in the top third. And the high-level cited papers in test set whose papers are all published in 2015 are defined as papers whose citations accumulated within five years rank in the top third.

Finally, considering that the one-third division criterion may cause papers with the same citation frequency are classified into different categories due to the total count of papers in the dataset. In order to avoid this phenomenon, this study adopts a downward compatible method to deal with the classification boundary problem, that is, if a certain citation frequency is classified as high-level, then all papers with the same citation frequency are high-level cited papers.

**Fig 1:**
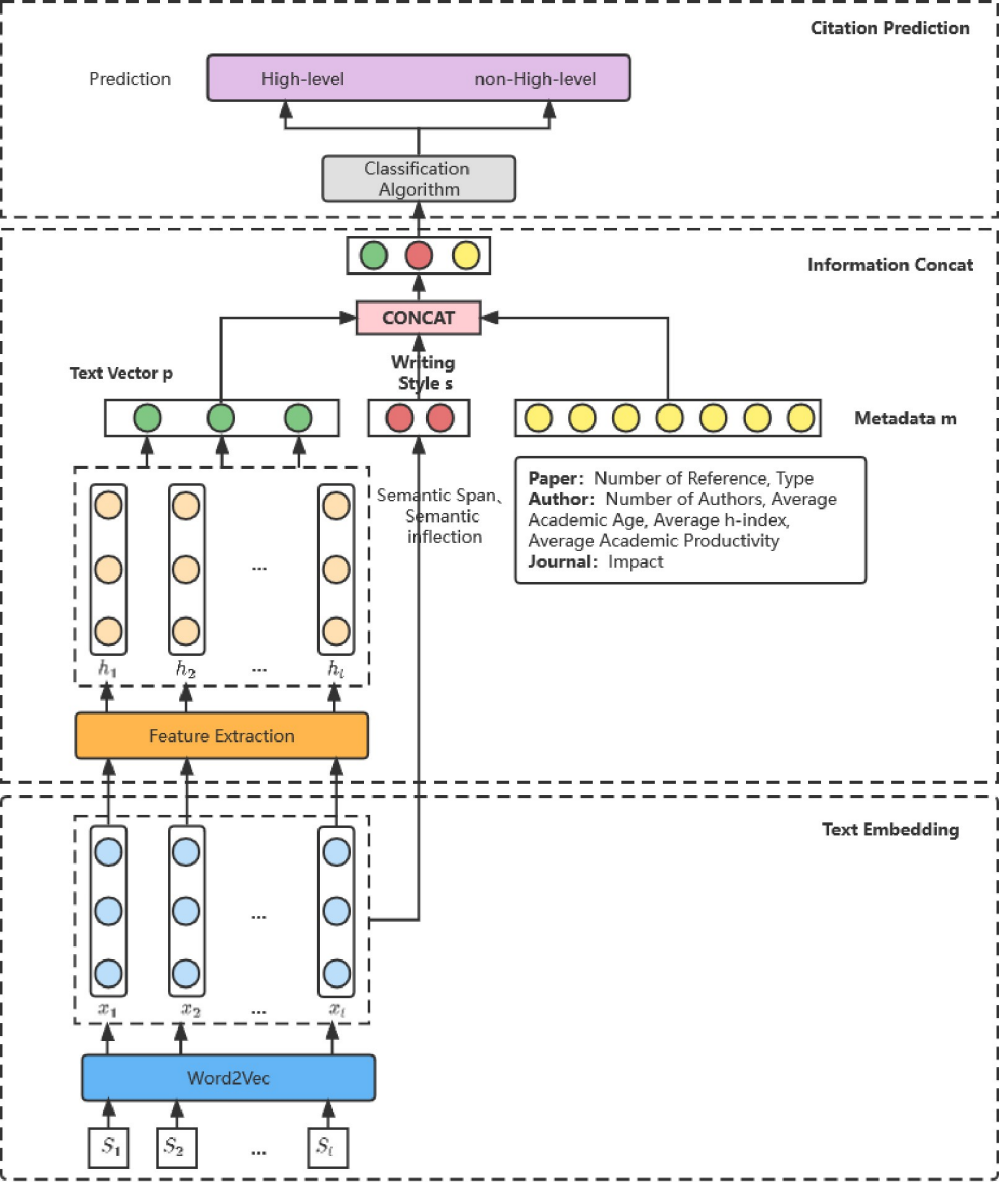
The framework of this research. Firstly, papers’ title and abstract are mapped to vectors. Secondly, the writing style index s is calculated. Then, the paper vector is encoded to form a text vector p. Finally, text vector p, style s, and metadata m are combined to predict citation potential.

In order to figure out whether the input information of this study is effective, we utilize machine learning methods such as SVM (Support Vector Machine) and Decision Tree to prove respectively. Artificial neural network is one of the most powerful and effective methods for classification learning, so we also designed a special neural network as the main component of our proposed method. Fig 1 illustrates the model proposed in this research that works best in the final experiment. Firstly, the papers’ title and abstract are mapped to the vector space, so that each paper has a corresponding vector representation; secondly, the paper writing style index *s* is calculated through the paper vector; Then, the paper vector is encoded by neutral network to form a text vector *p* by feature extraction. Finally, the text vector *p*, writing style *s* and metadata *m* are combined to predict the cited potential of papers.

### 3.3 Implementation details

According to the Price Index, the literatures can be divided into two categories, one of which is "the literature with current effect", which refers to the literature that has been published for less than five years [44]. The task to predict papers’ citations includes predicting the cumulative citations of papers and the annual citations of papers, and the cumulative citations of papers can be defined as regression model and classification model. The regression model refers to using the relevant features of the paper to predict the specific citation frequency in a certain time period in the future [45], which is an ideal prediction method. However, due to the finer prediction granularity, the error of the prediction result is larger. Therefore, more and more researches define the paper citation prediction task as a classification task. For example, papers are divided into two categories based on whether they increase the influence of the author after their publication [46]. If the citations of the paper can exceed the H-index of its author, it means that this paper helps to improve the influence of its author. Bhat et al. and others directly classify their datasets into three categories according to citations, they use approximate 33-rd and 66-rd percentiles of the citation distribution to form three classes [47]. Since the citation frequency prediction is defined as a classification problem, the prediction granularity is coarser, and the trained model has better generalization ability, and the research results are more valuable in practical applications. Therefore, in the follow-up research, more and more studies adopt to define paper citation prediction as a classification problem [47, 48]. In this research, citation prediction task is also defined as classification problem. The definition of highly cited paper is elaborated in the Implementation Progress. And this study mainly considers the rank of paper’s citations as the classification standard for whether a paper’s citations can become high-level or not.

As the Fig 1 shows, the first step is text embedding. We employ Word2Vec to extract semantic features from metadata sentences. Word2Vec is a neural probabilistic language model based on the distributional hypothesis, which states that words with similar contexts have similar semantic meanings. The training objective of Word2Vec algorithm is to maximize the average log probability given the sequence of words w(t-n), ⋯, w(t-2), w(t−1), w(t+1), w(t+2), ⋯, w(t+n):

$$L=\sum_{w\in C}\;\log\;p(w(t)|context(w(t)))$$ (1)

**Fig 2:**
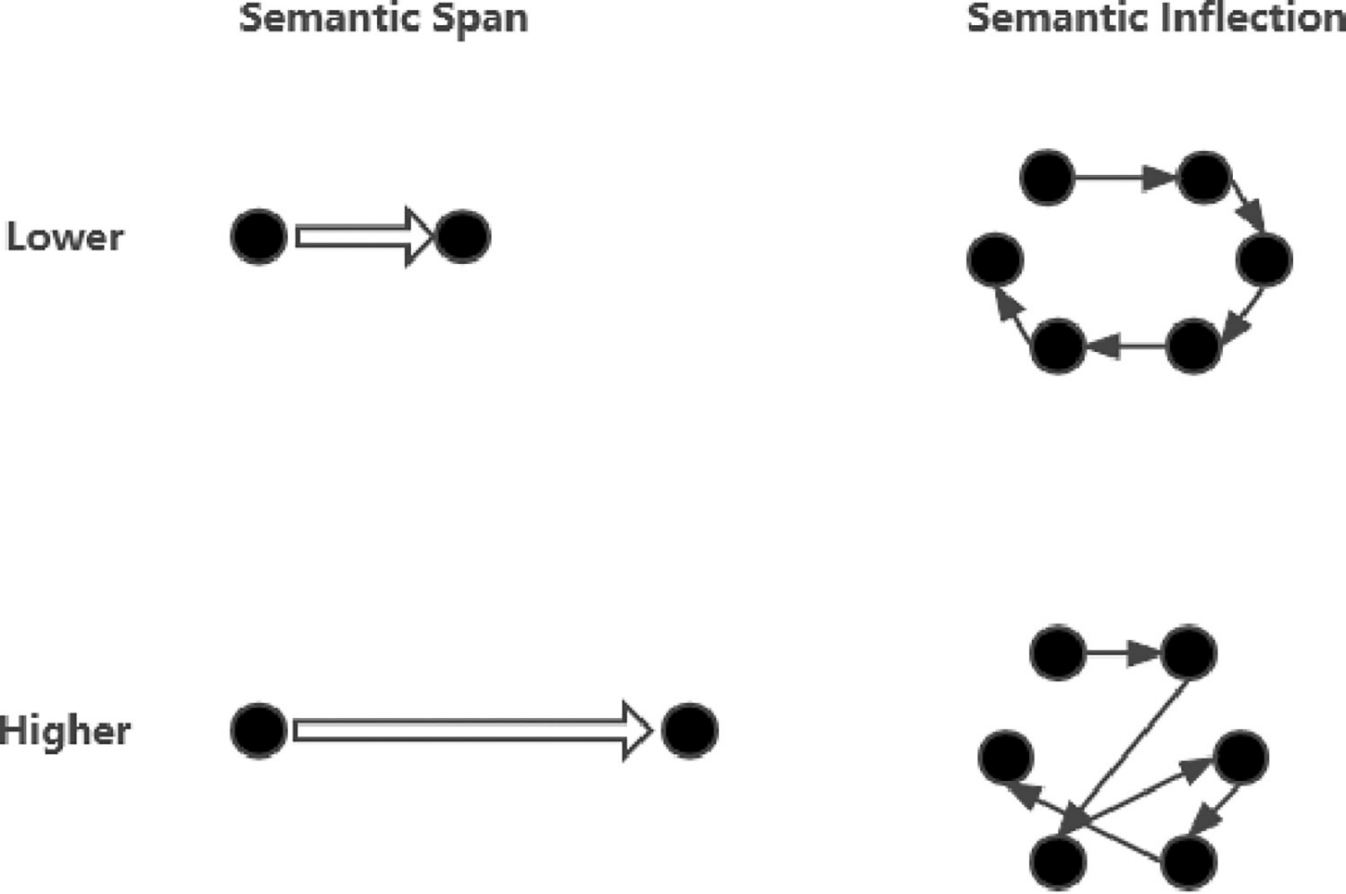
Example of semantic span and semantic inflection. Semantic Span reflects the amount of information contained in the sentence. Semantic Inflection reflects the amount of information contained in the sentence.

This research integrate title, abstract, writing style and metadata to predict whether a paper’s citation could be high-level. And to quantify writing style, we use two index, semantic span and semantic inflection (Fig 2).

Semantic span is a measure of the semantic difference between two sentences. The larger the semantic span is, the farther the meanings expressed by the two sentences are. Obviously, the larger the semantic span, the more semantic information the text contains, but at the same time, the higher the requirements for the reading ability of the readers who read the text will be [49] (McNamara er al., 1996). The semantic span of text is calculated as follows:

$$Semantic\;Span=\frac{\sum_{t=1}^{T-1}\;distance(t)}{T-1}$$ (2)

where T is the total number of sentences the text contains, and distance(t) is the cosine similarity between consecutive sentences.

The semantic inflection reflects the presentation order between different sentences. In this study, each sentence in the abstract is regarded as a point in the undirected graph, and the semantic span between sentences is used as the weight of the undirected graph edge. The semantic inflection degree is the ratio of the actual path length of the sentence to the length of the shortest path passing through all points. For example, if the text contains (SE_1_,SE_2_,…,SE_T_) a total of T sentences, the actual path of the sentence is

$$Actual=\sum_{t=1}^{T-1}\;distance(t)$$ (3)

The shortest path passing through all points is defined as the shortest path starting from SE_1_, ending at SE_T_, and passing through (SE_2_,SE_3_,…,SE_T-1_). thus,

$$Semantic\;Inflection=\frac{Actual}{shortest\_path}$$ (4)

The selection of metadata is based on the following (Table 2). First, the metadata related to the paper includes early citations, research topics, references, titles, abstracts, keywords, paper types and subject areas, etc. In this study, the early citations of papers are the target to predict, the subject area is limited to the field of artificial intelligence, and the research topic, paper title, paper abstract and keywords are included in the paper semantic information module, so the paper related metadata finally selects the paper type and references. Second, metadata related to author mainly includes the author h-index, the number of published papers, the number of authors, and the author’s age. In this study, the author’s academic age is used instead of the author’s age. Therefore, our research chooses the number of authors, the average academic age of the author, the average h-index of the author, and average academic productivity. Final, journal-related metadata includes journal impact factor and citation frequency. Scopus provides CiteScore as a substitute for journal impact factor. Therefore, journal citation score is selected as journal-related metadata.

**Table 2:** Selection of metadata.

Type	Metadata
Paper	Paper Type, Number of Reference
Author	Number of Authors, Average Academic Age, Average h-index, Average Academic Productivity
Journal	CiteScore

Inspired by previous work [7], we use algorithm including SVM, Decision Tree, CNN and LSTM to predict paper’s cited potential.

SVM maps the input vector x to a high-dimensional feature space z by means of a pre-selected mapping function, and it constructs an optimal hyperplane in this space that can maximize the separation boundary between different classes of samples [50].

Decision Tree is an algorithm of learning the conditional probability distribution of the output Y given the input variable X. In this study, we adopt one type of decision tree: Classification and Regression Tree (CART). CART algorithm uses binary tree to judge whether the input meets certain conditions, resulting in only two choices of "True" and "False", and finally the decision tree is obtained. CART algorithm is mainly composed of decision tree generation and pruning.

**Fig 3:**
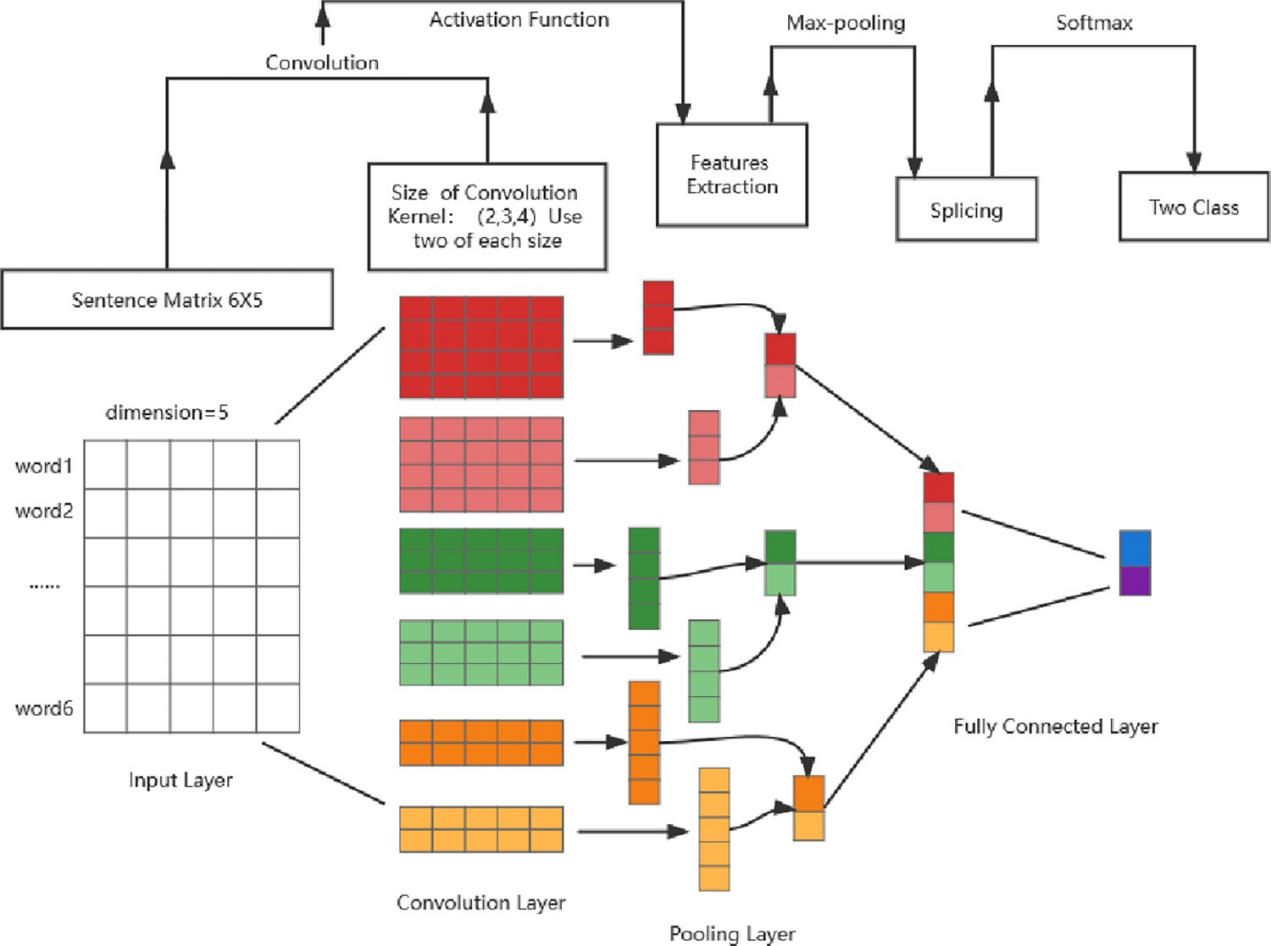
The convolutional neural network.

CNN is composed of input layer, convolution layer, pooling layer and fully connected layer (see Fig 3). The function of input layer converts is word embedding, initializing the input text with the word vector, and obtaining the vector representation of the input text as the input of the neural network. Convolution layer carry out feature extraction. Text features were extracted by convolution kernel. The pooling layer samples the outputs of the convolution layer respectively and splices the sampling results to obtain the text representation vector after the final feature extraction. After pooling, CNN get a vector whose dimension is the same as the number of convolution kernels. The full-connection layer maps the outputs of the pooling layer to a vector whose dimension is equal to the number of categories contained in the data set. Then Softmax function output the prediction probability of samples belonging to each category. The class with the highest probability is taken as the category of sample belonging, and the text classification is finally completed.

**Fig 4:**
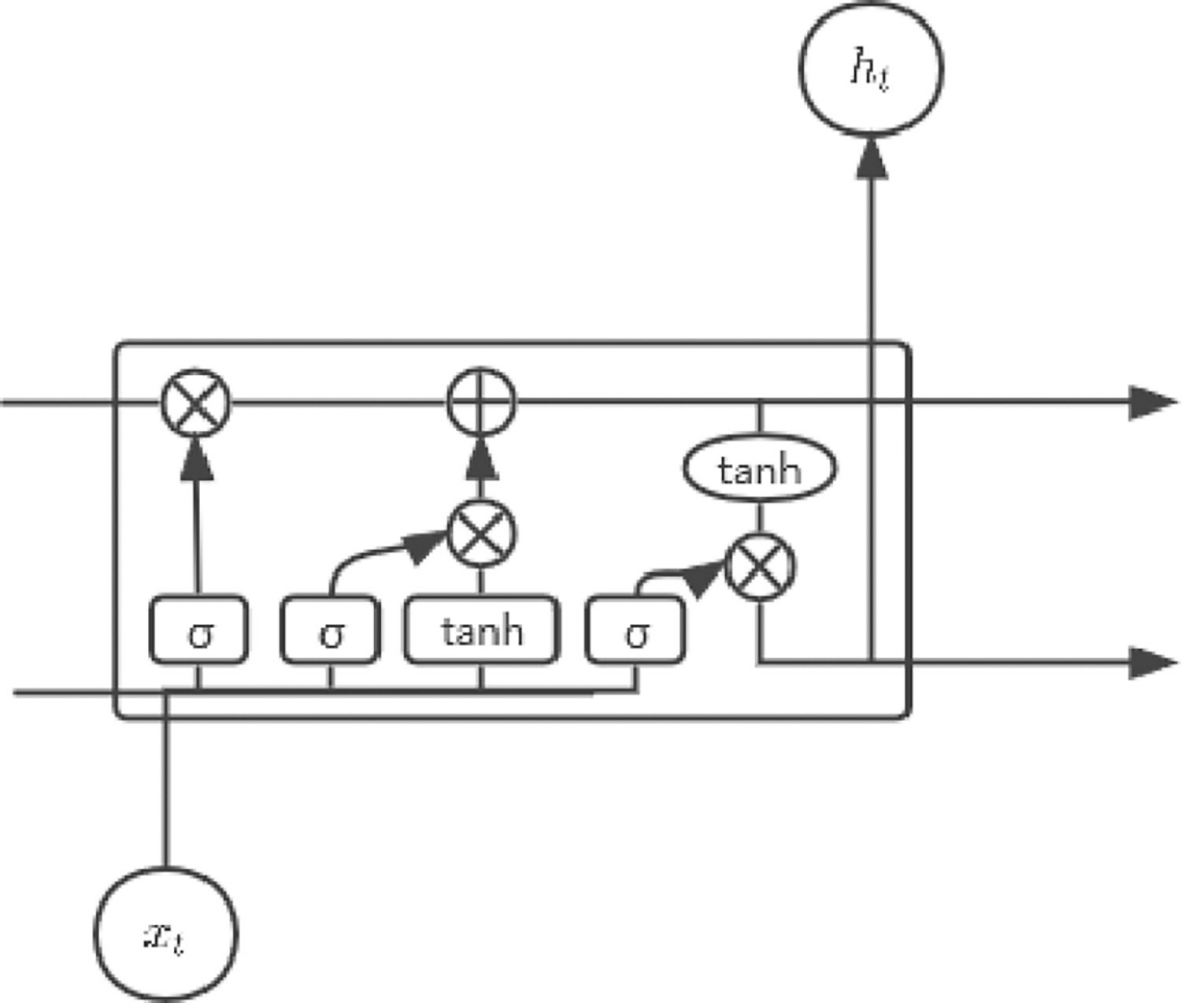
Long short-term memory network.

LSTM is a kind of recurrent neural network, the core is the cell state (see Fig 4) [51]. LSTM is firstly to decide which information to discard and which to retain. This step is achieved through the forget gate. The forget gate checks the cell state of the previous LSTM unit and current input, and determines the information to be discarded through the Sigmoid function whose output value is between 0 and 1. The closer to 0 means discarded, and the closer to 1 means retained. The second step is to decide what information to store in the LSTM cell. There are two parts to this step. First, the Sigmoid function through the input gate determines which values to update, and then the candidate cell state is created through a tanh function. The third step is to update the cell state, adding the linear change of the output state value of the first step and the second step as the updated cell state. Finally, it is decided to determine the hidden layer state of the current LSTM unit, which is realized through the output gate. The output gate uses the Sigmoid function to determine which parts to output, and multiplies the cell state mapped by the tanh function to get the final hidden layer state as the final output of the LSTM cell.

Table 3 describes the models’ parameters which resulted in the best outcomes based on our experiments. Such implementation details are worth noting because designing an effective classification algorithm is a challenging task for this application which requires investigating many design choices. We use model of CBOW to extract semantic features which is one of model of Word2Vec. Then for SVM and Decision Tree, we carry out Rbf kernel function of SVM and set the max depth of the Decision Tree to 4. In addition, we utilized “Keras” framework (https://keras.io) which is a well-known and widely used implementation for artificial neural networks and deep learning techniques. In addition, we used the “CNN” and “LSTM” module of Keras for implementing the proposed neural network. In order to avoid overfitting, the “Dropout” technique is used with the rate of 0.2. This technique enables more epochs (20 epochs in our experiments) in the training phase while minimizing the risk of overfitting [52]. We implemented the Adam optimization algorithm, which is an effective method for training neural networks, with the initial learning rate of 10–3. Data are fed to the network in batched 512 papers(batch-size = 512). Table 3 summarizes the implementation details of the proposed method.

**Table 3:** The implementation details of the proposed method.

Module	Parameters	Value	
Text Embedding	sg	0	
	vector_size	100	
	alpha	0.025	Word2vec
	cbow_mean	1	
	min_alpha	0.0001	
Classification Algorithm	kernel	Rbf	
	C	0.5	SVM
	tol	0.001	
	criterion	Gini	
	splitter	Best	Decision Tree
	max_depth	4	
	min_samples_leaf	5	
	kernel_size	[3, 4, 5]	
	units	128	
	class_weight	Balanced	
	learning_rate	0.001	
	batch_size	512	CNN&LSTM
	epochs_num	20	
	optimizer	Adam	
	loss	CrossEntropy	
	dropout	0.2	

After obtaining the text vector representation of the paper, the next step is to integrate the writing style, text vector and the metadata of the paper. Combining information from multiple sources can improve the accuracy of model predictions. In this study, the concatenate strategy is used to integrate information of text vectors, writing styles and metadata. concatenate directly connects the two features, that is, the amount of information contained in each feature of the sample remains unchanged, but the dimension of the vector describing the feature of the sample increases. For example, if the dimensions of the two input features x and y are p and q, the dimension after adopting the concatenate strategy is p+q.

## 4. Data

**Fig 5:**
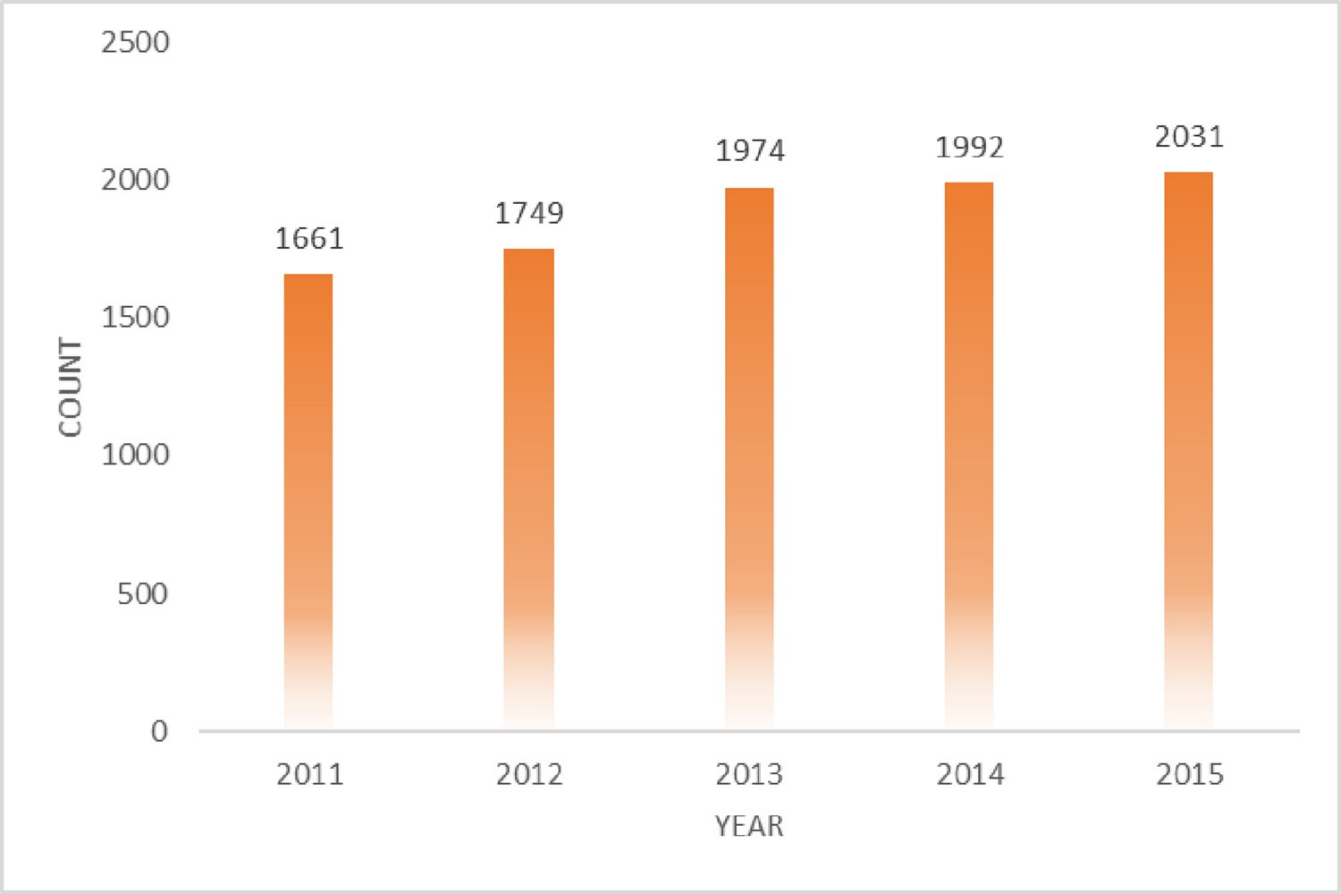
Number of papers per year. The average number of articles per year is around 1800.

**Fig 6:**
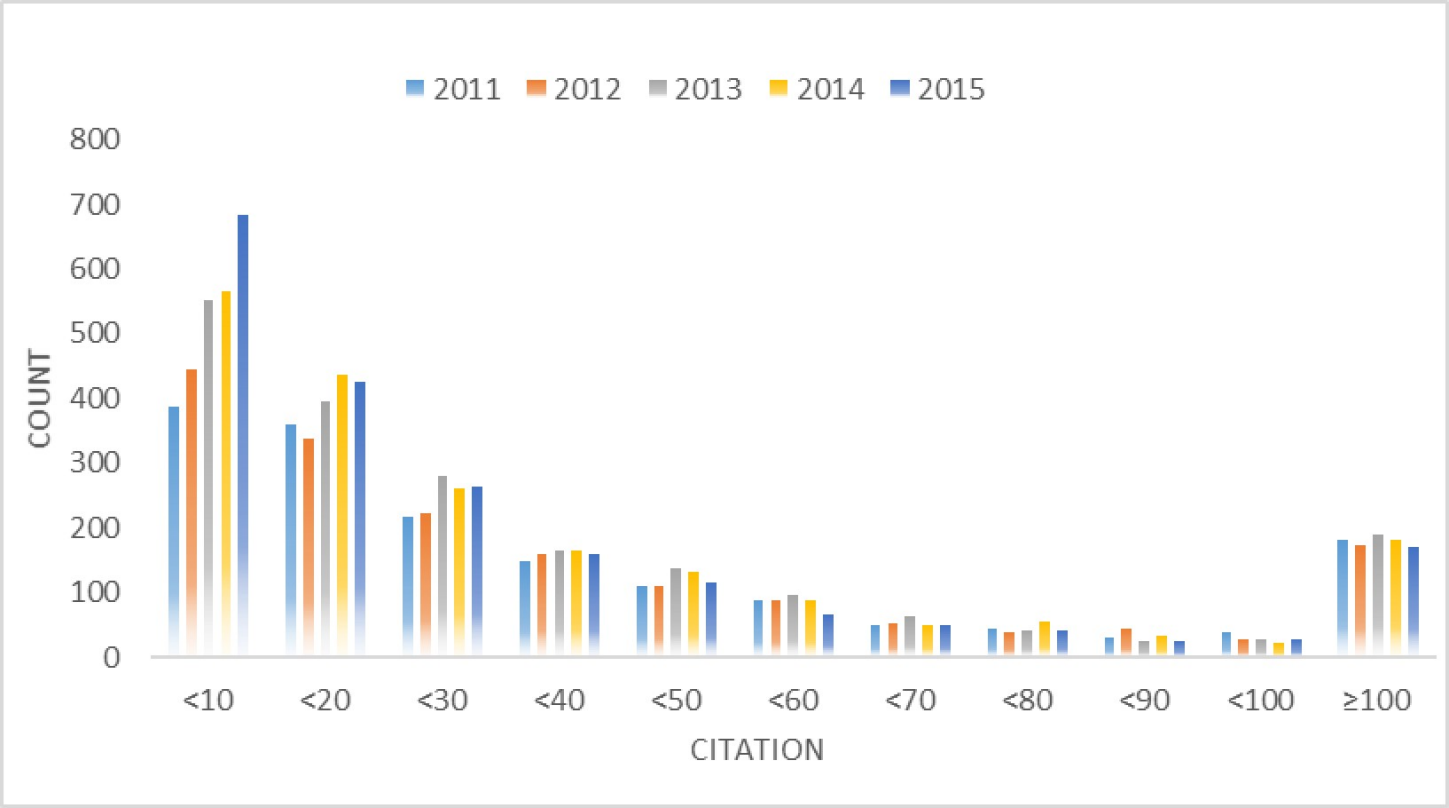
Interval distribution of citations. The vast majority of papers are cited less than 10 times.

In recent years, the field of artificial intelligence has developed rapidly, and its research results have been increasingly applied in daily life. Hence, we selected a total of 23 journals at A, B levels in the field of Artificial Intelligence from China Computer Federation catalogue 2019, they are Artificial Intelligence (AI), IEEE Trans on Pattern Analysis and Machine Intelligence (TPAMI), International Journal of Computer Vision (IJCV), Journal of Machine Learning Research (JMLR), ACM Transactions on Applied Perception(TAP), Autonomous Agents and Multi-Agent Systems (AAAMS), Computational Linguistics, Computer Vision and Image Understanding (CVIU), Data and Knowledge Engineering (DKE), Evolutionary Computation, IEEE Transactions on Affective Computing(TAC), IEEE Transactions on Audio, Speech, and Language Processing (TASLP), IEEE Transactions on Cybernetics, IEEE Transactions on Evolutionary Computation (TEC), IEEE Transactions on Fuzzy Systems (TFS), International Journal of Approximate Reasoning (IJAR), Journal of Artificial Intelligence Research (JAIR), Journal of Automated Reasoning, Journal of Speech, Language, and Hearing Research (JSLHR), Machine Learning, Neural Computation, Neural Networks respectively. And we consider using the widely-used Scopus database which is a scientific literature database to obtain the required data in our experiment. Scopus is one of the largest literature and citations database in the world. We choose the pybliometrics python library developed by Rose and Kitchin [53] to extract information of metadata such as title, abstract and so on. The final dataset includes 9407 papers published from 2011 to 2015 (see Fig 5). In our dataset, most papers’ citation is distributed in the range of 0–30 (see Fig 6).

In Experiment 1, we select papers published in the field of artificial intelligence between 2011 and 2015 are selected. The primary reason is for this choice is that the research hotspots will evolve over time, and the time span can significantly affect the impact of paper semantics on citations. We considered the top third of the citations of the papers to be high-level cited papers. The size of the training and test sets, along with the number of high-level cited and non-high-level cited papers, are summarized in Table 4.

**Table 4:** Dataset of the Experiment 1.

	Training-set	Test-set	Total
Total	7524	1883	9407
High Level	2814	718	3532
Non-High Level	4710	1165	5875

Experiment 2 mainly uses the papers published from 2011 to 2013 as the training set, and the papers published in 2015 as the test set. The training and test sets, along with the number of high-level cited papers and non-high-level cited papers, are detailed in Table 5.

**Table 5:** Dataset of the Experiment 2.

	Train	Test	Total
Total	5384	2031	7415
High Level	2145	659	2804
Non-High Level	3239	1372	4611

## 5. Result

### 5.1 Measurement criteria

Commonly used classification model evaluation indicators are usually calculated according to the confusion matrix, as shown in Table 6.

**Table 6:** Confusion matrix.

True class Hypothesized class	positive	negative
**positive**	TP	FP
**negative**	FN	TN

**(1) Accuracy.** Accuracy refers to the ratio of the number of correctly predicted samples to the predicted samples by the model to the total number of predicted samples. The calculation formula is shown in Formula (5).

$$Accuracy=\frac{TP+TN}{TP+FP+TN+FN}$$ (5)

**(2) F1-score.** F1-score is a comprehensive index considering confusion matrix, the calculation formula is shown in Formula (6).

$$F1-score=\frac{2*TP}{2*TP+FP+FN}$$ (6)

The value range of the above two classification evaluation indicators is within [0, 1]. In general, the larger the F1 value and accuracy rate, the better the model performance. Accuracy is usually suitable for evaluating datasets with approximately equal numbers of categories. The F1 value can comprehensively consider the recall and precision of the model. When comprehensively considering precision and recall rates, the F1 score is commonly used. In this study, the accuracy and F1-score are employed as our evaluation index [54, 55].

### 5.2 Result

Table 7 shows the results of experiment 1.

**Table 7:** The experimental results of citation comparison prediction.

		F1-score	Accuracy
Title, Abstract	SVM	0.2356	0.6623
	Decision Tree	**0.5888**	**0.724**
Title, Abstract +Writing Style	SVM	**0.7353**	**0.7952**
	Decision Tree	0.6963	0.7672
Metadata	SVM	0.7855	**0.841**
	Decision Tree	**0.7936**	0.8315
Title, Abstract + Writing Style + Metadata	SVM	0.7865	0.8433
	Decision Tree	0.7996	0.84
	LSTM	0.8542	0.8804
	CNN	**0.8913**	**0.8929**

From Table 7, we can see that the information of title and metadata can predict the impact level of academic paper respectively. And writing style helps to improve the performance of the proposed model. In addition, the accuracy of integrating title, abstract, writing style and metadata up to 0.89, which outperforms methods that take single information as input. So the experiment 1 proves that it is reasonable to predict cited potential of academic paper by the paper’s semantic information and metadata.

From the results of experiment 1, it can be found that it is reasonable to use the paper metadata and the semantic information of the paper to predict the cited potential, and the accuracy of integrating title, abstract, writing style and metadata up to 0.89. However, the experimental results of Experiment 1 are relatively limited in practical applications, so Experiment 2 uses instant prediction experiments to verify the effectiveness of using paper metadata and semantic information for paper citation prediction. The model evaluation indicators selected in experiment 2 are consistent with those in experiment 1. In addition, Experiment 2 selects the classification algorithm with better output effect from Experiment 1. The results are shown in Table 8:

**Table 8:** The experimental results of citation instant prediction.

		F1-score	Accuracy
Title, Abstract + Writing Style + Metadata	SVM	0.7172	0.8421
	LSTM	0.8161	0.8837
	CNN	**0.8472**	**0.8847**

From the Table 8 we can see that, the maximum accuracy of the proposed model is up to 0.88, which means that it is effective in practical applications to predict the cited potential using semantic and metadata information.

## 6. Summary

Aiming to predict paper’s potential to become high-level cited, this paper proposes a technical framework for predicting high citation potential. Based on review existing research, we have identified key indicators that influence a paper’s citation potential, including metadata, title, abstract, and writing style. Subsequently, we developed a deep learning-based predictive model to recognize high citation potential papers. Our predicting framework demonstrates promising predictive performance in classification experiments and case studies, indicating the efficacy of the proposed method.

Furthermore, we have reached the following conclusions. First, both the content information and metadata of a paper play significant roles in predicting its cited potential. The predicting experiments with either content information or metadata alone as the input achieves an accuracy of over 0.7. Notably, using metadata alone for citation frequency prediction yields a higher accuracy compared to using content information alone, with an accuracy exceeding 0.8. Second, integrating semantic information such as the textual content and writing style with the paper’s metadata can significantly enhance the prediction performance. The model combining semantic information with metadata, outperforms the use of either type alone, achieving a final prediction accuracy of 0.89.

## 7. Discussion

The existing researches on the citation potential of papers cannot overcome the shortage of delayed prediction that existed citation data of the paper is needed. In order to optimize the process and evaluate the potential impact of academic papers accurately, this paper proposes a model which combine paper’s metadata and semantic information to predict whether a paper can become high-level cited paper instantly. In the case study, we use papers published between 2011 and 2013 to predict cited potential of papers published in 2015. The overall accuracy of the best model exceeds 0.88, thereby demonstrating the practical effectiveness of instant prediction of cited potential using metadata and semantic information. And the results of experiments demonstrate that the proposed model can instantly predict high-level cited papers, and it is reasonable and effective in practical applications.

The experiments also indicate that metadata plays a predominant role in predicting the impact of papers. For instance, papers published in high-impact factor journals and those authored by well-known researchers often exhibit higher academic influence. This is attributed to journal and author-related factors. However, the content of the paper also plays a crucial role in enhancing the predictive performance of the model, such as the paper’s title and abstract. This is because elements like the title and abstract to some extent reflect the paper’s innovativeness and academic value. Indicators extracted from the text, such as semantic inflection and semantic span, further enhance the semantic information within the text and abstract. Integrating these data offers a potential avenue for identifying the value and potential impact of new academic papers. The model proposed in this paper successfully achieves this goal.

Furthermore, even if the identified high-level cited papers are not actually high-level cited, they may still receive more citations than other papers. There is a boundary in this paper for determining whether a paper is high-level cited. Papers with citation frequency in the top one-third of the dataset are considered high-level cited. However, frequency of being cited are a relatively continuous measure. The definition in this paper may result in cases where papers with similar citation frequency are categorized differently. In other words, the boundary between high cited and non-high cited papers is rather ambiguous, making feature learning challenging for the model. Addressing this issue could further enhance the effectiveness of the proposed predictive framework. This also means that this paper will have a good performance in recognizing the paper with a large amount of cited frequency. Because the recognized paper may rank at the boundary of the high cited and the non-high cited. The paper can still attract many citations although its citation frequency can’t reach the standard of high-level cited paper.

However, this research still has some limitations. Firstly, the dataset used by the model is only focused on the field of artificial intelligence, and its effectiveness in other fields remains to be verified. Secondly, the semantic part uses the title and abstract instead of the full text, which cannot fully represent the semantic information contained in the paper. As the future works, we will consider more field to verify the model’s performance and use full text as the semantic inputs instead of title and abstract. We will also intend to apply Transformer-based models, such as RoBERTa [56] to enhance the prediction effectiveness of the model.
